# Supplementary material for: A Bio-Inspired Chaos Sensor Model Based on the Perceptron Neural Network: Machine Learning Concept and Application for Computational Neuro-Science
Source: Sensors (Basel). 2023 Aug 12;23(16):7137. doi: 10.3390/s23167137 (PMC10458403; doi:10.3390/s23167137)
Supplement: Supplementary file 1 [file sensors-23-07137-s001.zip › Model_1/Model_1 data sheet.pdf]

Model_1 data sheet

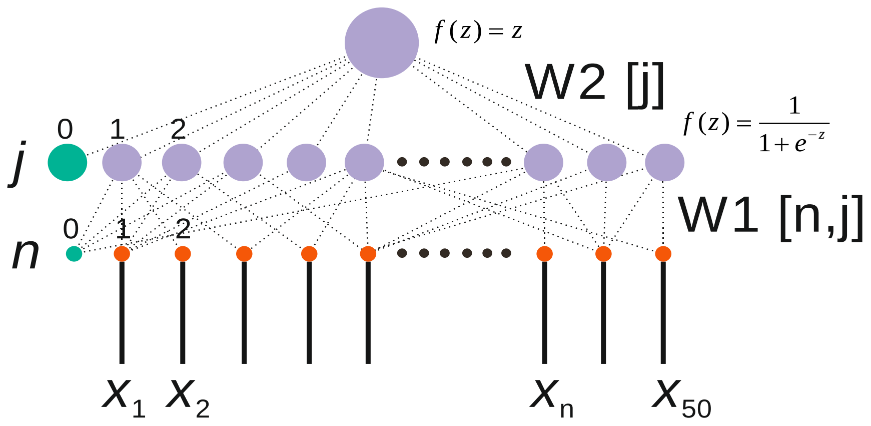

Figure 1.

Figure 1 shows a perceptron model with the number of neurons in the hidden layer *NH* = 50.
The input is a time series x_n
Preliminary normalization of series was performed by subtracting the *Mean* = 32.28749 value from the values of the series elements.
Input series from datasets Base_1, Base_2 or Base_1_2
Files:
coeffs_1_50x50.txt - Weights W1[n,j]; n=1…50, j=1…50.
Data format:
W1[1,1] W1[1,2] W1[1,3]…
W1[2,1] W1[2,2] W1[2,3]…
…

inter_1_50x1.txt - Bias W1[0,j]; j=1…50
Data format:
W1[0,1]
W1[0,2]
…

coeffs_2_50x1.txt - Weights W2[j]; j=1…50
Data format:
W2[1]
W2[2]
…

inter_2_1x1.txt -Bias W2[0]
Data format:
W2[0]

The activation function is shown in the figure 1.
model.pkl Perceptron models trained on the Base_2 with the number of neurons in the hidden layer *NH* = 50, with preliminary normalization of time series (from the scikit-learn library saved with the pickle library https://docs.python.org/3/library/pickle.html)
